# Supplementary material for: Heterogeneity in VEGFR3 levels drives lymphatic vessel hyperplasia through cell-autonomous and non-cell-autonomous mechanisms
Source: Nat Commun. 2018 Apr 3;9:1296. doi: 10.1038/s41467-018-03692-0 (PMC5882855; doi:10.1038/s41467-018-03692-0)
Supplement: Supplementary file 1 — Supplementary Information(PDF 10430 kb) [file 41467_2018_3692_MOESM1_ESM.pdf]

Supplementary Information

Heterogeneity in VEGFR3 levels drives lymphatic vessel hyperplasia through cell-autonomous and non-cell-autonomous mechanisms

Zhang et al.

Supplementary Figures

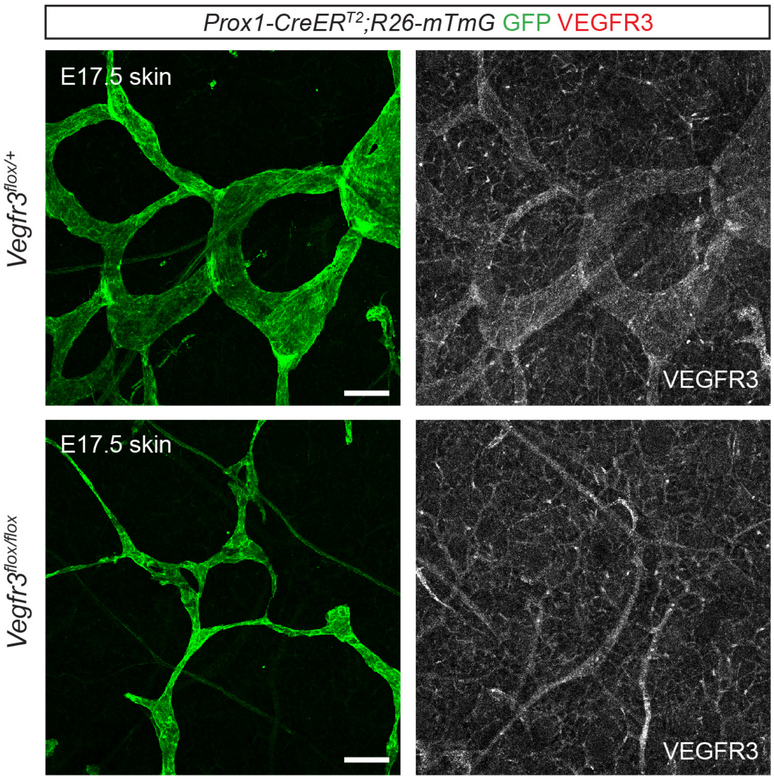

Supplementary Figure 1 | Efficient VEGFR3 protein depletion in dermal lymphatic vessels in the *Vegfr3^{flox};R26-mTmG;Prox1-CreER^{T2}* embryos. Whole-mount immunofluorescence of E17.5 embryonic skin. Cre activity was induced by 4-OHT (1 mg) administration to pregnant females on 6 consecutive days starting at E10. Scale bars: 100 µm.

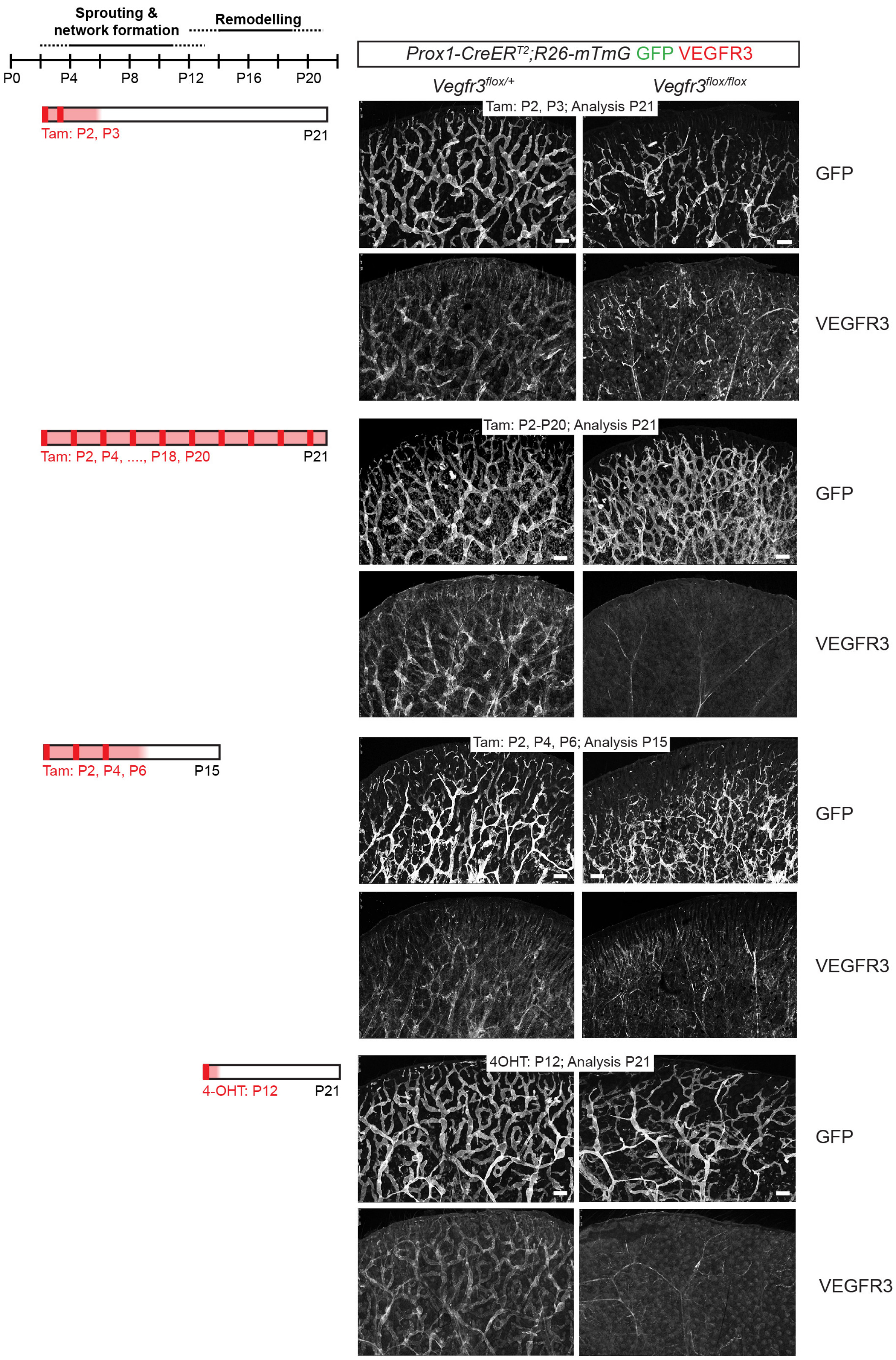

Supplementary Figure 2 | Time course analysis of the effect of postnatal *Vegfr3* deletion on dermal lymphatic development. Left: Tamoxifen administration schedule. Cre induction (n × 150 µg of Tam or 100 µg of 4-OHT) in red and expected effective period (72 h for Tam and 24 h for 4-OHT) in light red are shown. Right: Single channel images of whole-mount immunofluorescence of ear skin for GFP and VEGFR3 of indicated stages and Tamoxifen/4-OHT treatment regimes. Scale bars: 200 µm.

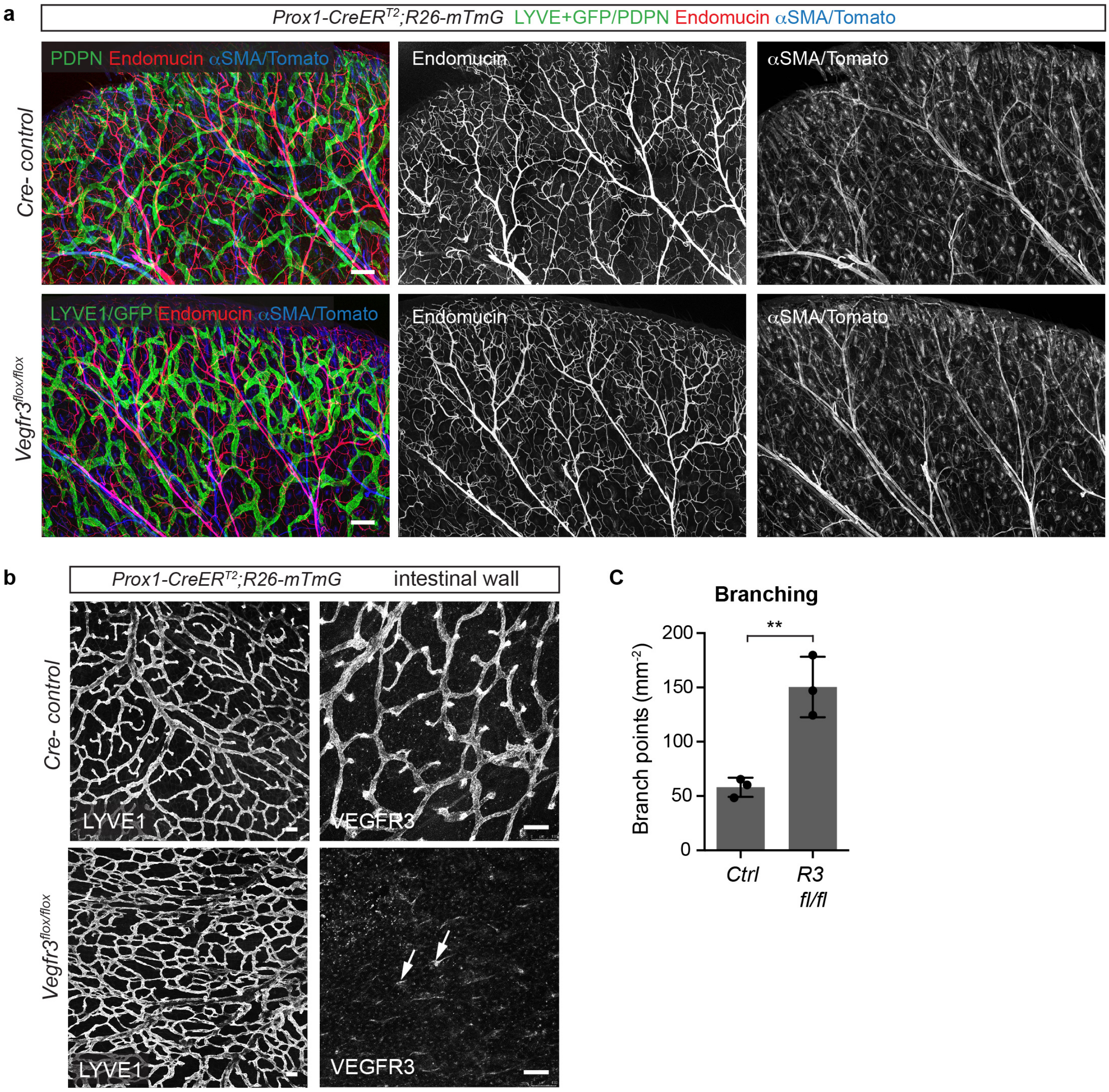

Supplementary Figure 3 | Analysis of the dermal blood and intestinal lymphatic vasculatures in the *Vegfr3^{flox};R26-mTmG;Prox1-CreER^{T2}* mice. Whole-mount immunofluorescence of ear skin (**a**) and intestinal wall (**b**) of 3 weeks old *Vegfr3$^{flox/flox}$;R26-mTmG;Prox1-CreER^{T2}* and Cre-negative littermate mice treated with Tamoxifen at P2, P4 and P6. Note normal dermal blood vasculature in mutant in comparison to control mice (**a**), visualized by staining with antibodies against Endomucin (showing veins and capillaries), and αSMA (smooth muscle cells) on the same channel with Tomato signal from the *R26-mTmG* reporter. Lymphatic vessels were visualized by staining for PDPN in Cre$^-$ controls, and for both LYVE1 and GFP in Cre$^+$ mutants. (**b**) shows hyperbranched intestinal lymphatic vasculature (left panels) and efficient depletion of VEGFR3 (right panels) except for residual non-targeted VEGFR3$^+$ cells (arrows) in the mutant. (**c**) Quantification of vessel branch points in lymphatic vasculature of the intestinal wall. Bars represent mean (n=3 mice) ± s.d. ** P<0.01. Two-tailed unpaired Student's *t*-test. Scale bars: 200 μm (**a**), 100 μm (**b**).

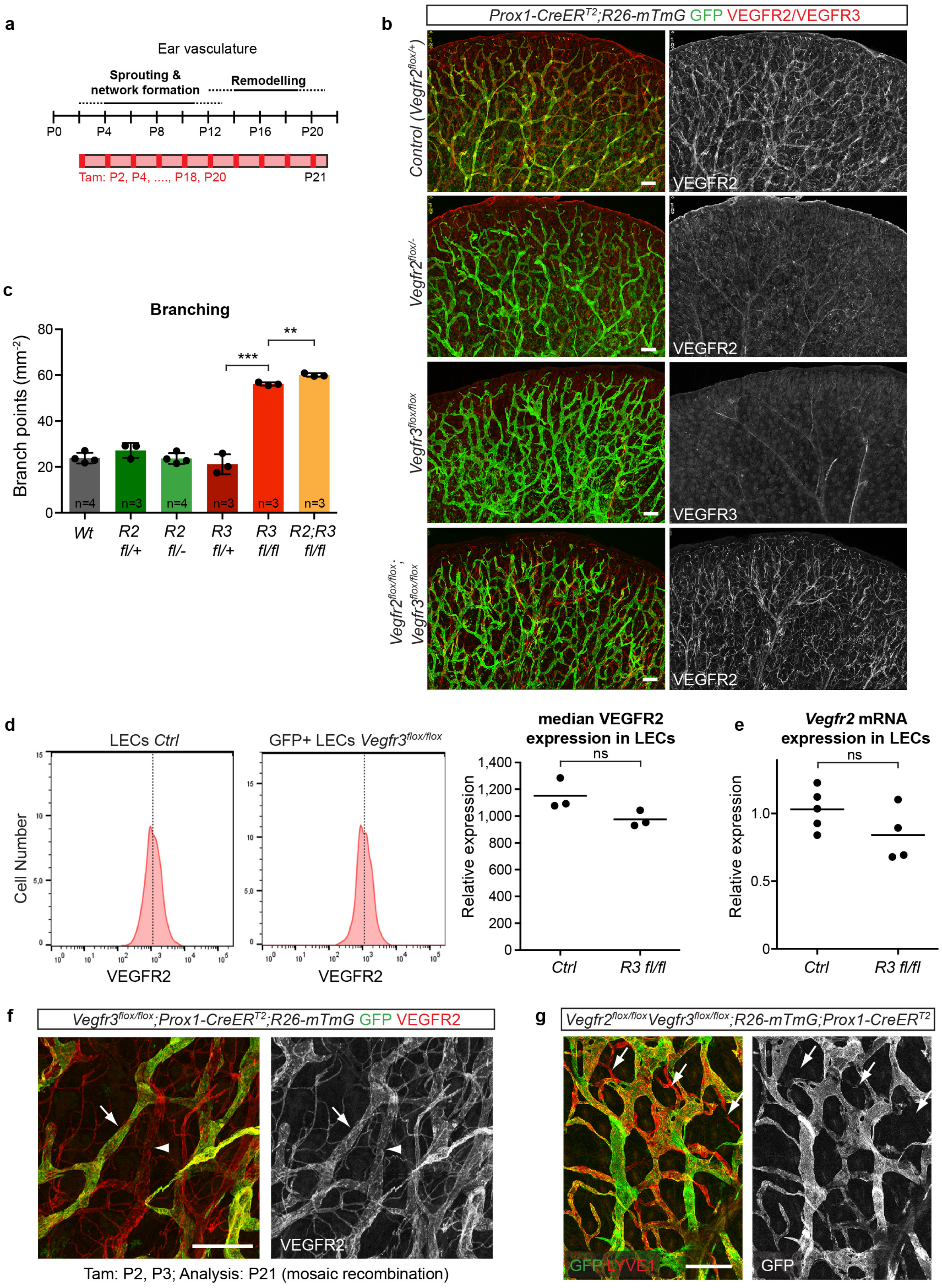

Supplementary Figure 4 | Analysis of the contribution of VEGFR2 to postnatal dermal lymphatic development and *Vegfr3* loss driven vessel hyperplasia. (**a**) Tamoxifen administration schedule (Cre induction, red (10 × 150 µg); and expected effective period (72 h), light red) and the timing of dermal lymphatic vessel formation in the ear. (**b**) Whole-mount immunofluorescence of ear skin of 3 weeks old mice of indicated genotypes. Single channel

images for VEGFR2 or VEGFR3 staining are shown. (**c**) Quantification of vessel branch points. Bars represent mean (n=3-4 mice, as indicated) ± s.e.m. Data for *Ctrl, R3 fl/+* and *R3 fl/fl* are presented again (from Fig. 2e) for comparison. (**d**) Representative FACS histogram of VEGFR2 expression in GFP^+ LECs (gated based on co-expression of PECAM1 and PDPN) from P9 *Vegfr3*$^{flox/flox}$*;R26-mTmG;Prox1-CreER*^{T2} mice compared to a Cre^- littermate. Dotted line displays median. Graph on the right shows median expression in littermate (Cre^- (n=2) and Cre^+ *R3 fl/+* (n=1)) controls (*Ctrl*) and mutant (*R3 fl/fl,* n=3) mice, horizontal line represents mean. (**e**) *Vegfr2* mRNA expression in sorted LECs from P13-P14 *Vegfr3*$^{flox/flox}$*;R26-mTmG;Prox1-CreER*^{T2} mice and Cre^- controls. Horizontal line represents mean (n=5 *Ctrl* and n=4 *R3 fl/fl* mice with n=2 technical replicates for each). (**f**) Whole-mount immunofluorescence of ear skin of 3 weeks old mice. Note similar VEGFR2 expression in targeted GFP^+ (i.e. $VEGFR3^-$) (arrow) and non-targeted GFP^- (i.e. $VEGFR3^+$) (arrowhead) vessels. (**g**) Whole-mount immunofluorescence of *Vegfr2*$^{flox/flox}$*;Vegfr3*$^{flox/flox}$*;R26-mTmG;Prox1-CreER*^{T2} ear skin showing vessel interconnections (arrows) formed of non-targeted (GFP^-) $LYVE1^+$ LECs. ** *P*<0.01, *** *P*<0.001, ns = not significant. Two-tailed unpaired Student's *t*-test (**c**, **d**, **e**). Scale bars: 200 μm (**b**, **f**, **g**).

Sort scheme for mouse LECs (P13-P14 ear skin)

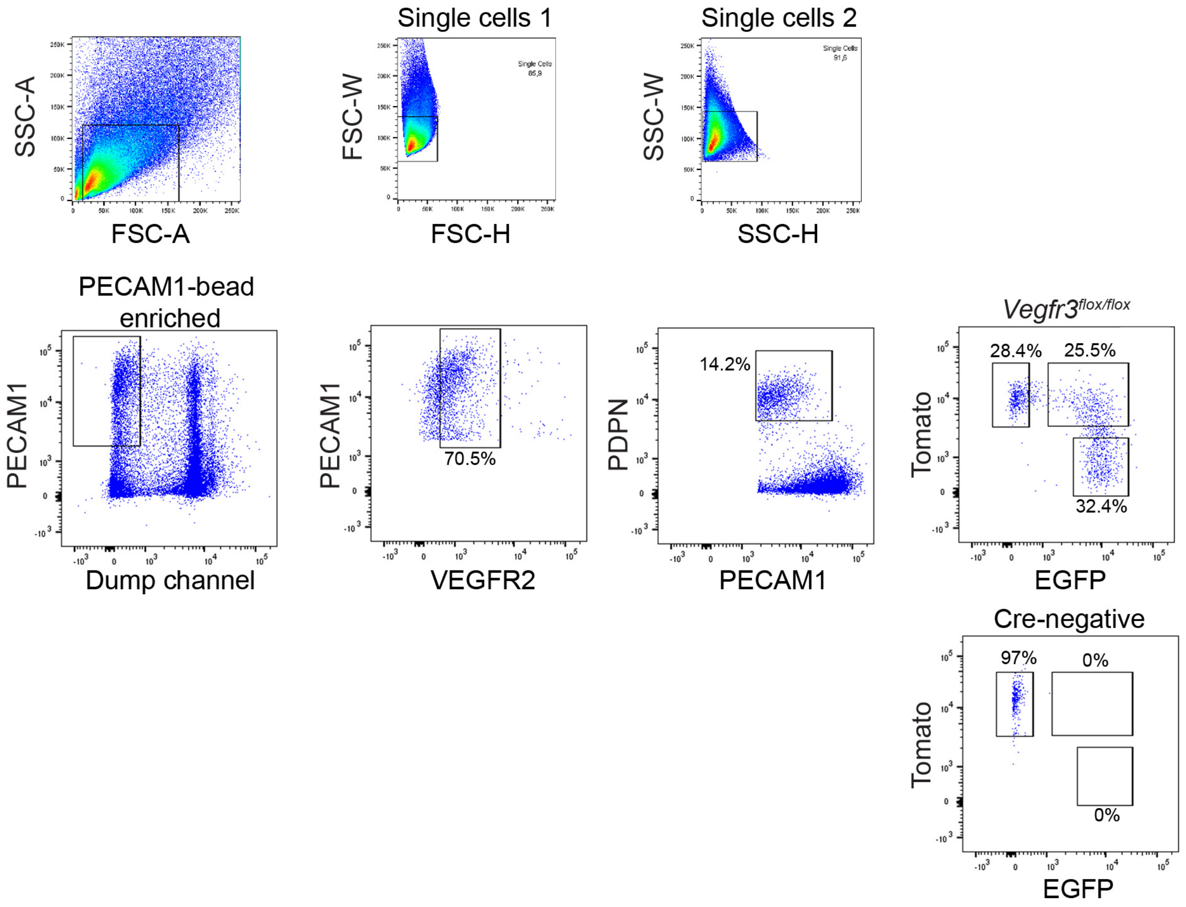

Supplementary Figure 5 | Isolation of dermal LECs by flow cytometry. Sort scheme for mRNA and genomic DNA analysis of LECs from *Vegfr3*$^{flox/flox}$*;R26-mTmG;Prox1-CreER*^{T2} and Cre^{-} control mice. ECs were enriched using CD31/PECAM1 magnetic microbeads. Single cells were gated using FSC-A/SSC-A followed by FSC-H/FSC-W and SSC-H/SSC-W. LECs were gated in three steps; 1. PECAM1^{high}, dump channel^{-} cells, 2. PECAM1^{+}, VEGFR2^{+} (ECs), 3. PDPN^{+} (LECs). LECs were subsequently sorted based on GFP and Tomato expression as indicated.

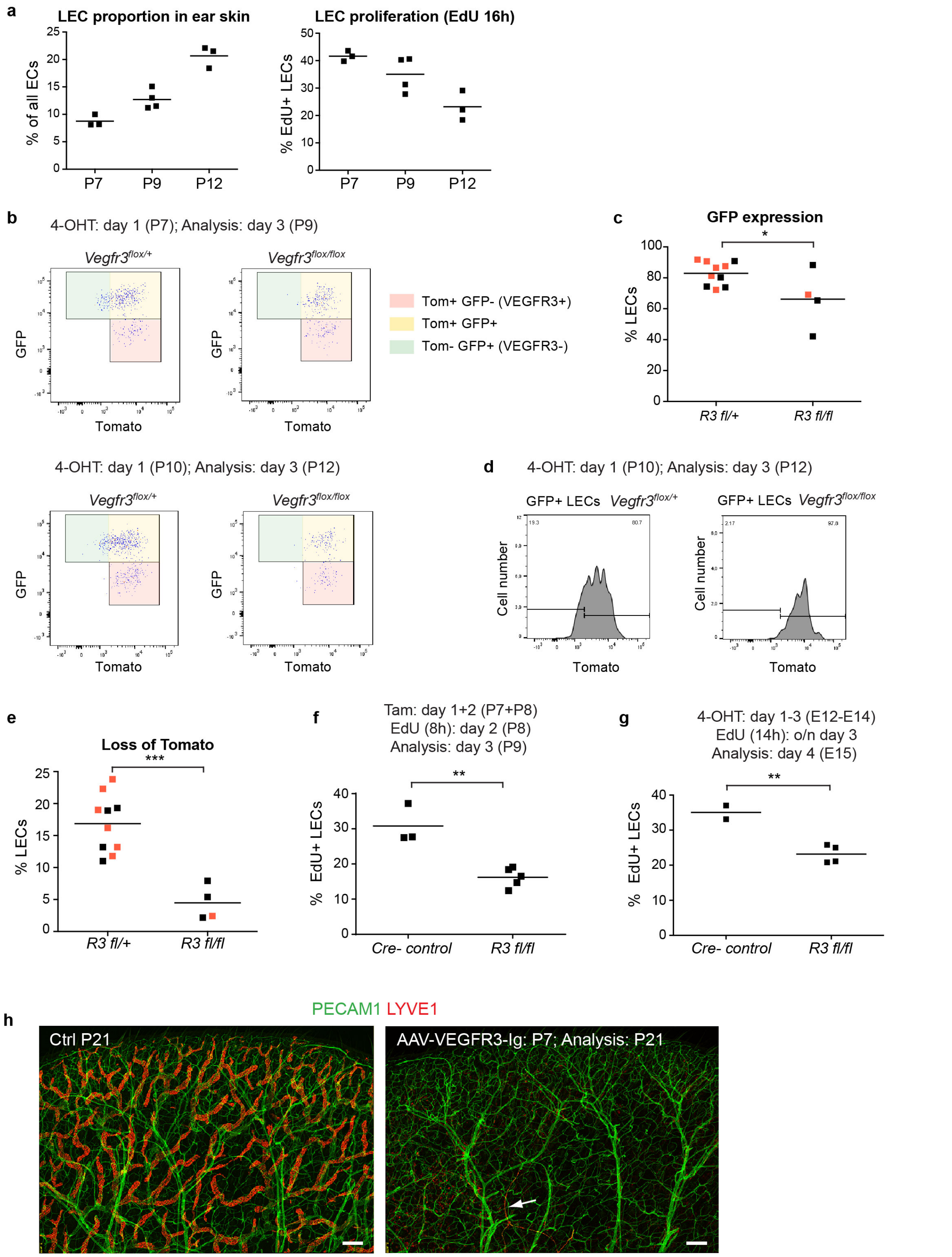

Supplementary Figure 6 | VEGFR3 contributes to the survival and proliferation of dermal LECs during early postnatal development. (**a**) LEC proportions (expressed as % PDPN$^+$ ECs (LECs) of all PECAM1$^+$ ECs) and proliferation (EdU incorporation for 16 h) determined in Cre$^-$ Tamoxifen treated pups at P7, P9 and P12 by flow cytometry. Horizontal lines represent mean (n=3-4 mice). (**b-e**) Analysis of Cre-mediated recombination after short-term induction.

Ear skin from P9 (upper plots) or P12 (lower plots) *Prox1-CreER*^{T2} positive pups carrying heterozygous (n=10) or homozygous (n=4) *Vegfr3*^{flox} allele and the *R26-mTmG* reporter were analyzed 2 days after administration of a single i.p. injection of 50 µg 4-OHT. Representative dot plots (**b**, P9 and P12) and histograms (**d**, P12 only) are shown. Summary graphs show GFP (**c**) and Tomato (**e**) expression in P9 (red squares) and P12 (black squares) mice. Note similar recombination efficiency (GFP expression, **c**) but selective reduction of GFP^{+} LECs that have lost Tomato expression in the mutant (*R3 fl/fl*) LECs (**e**), as evaluated by percentage of GFP^{+} Tomato^{-} cells within the total GFP^{+} LEC population. (**f**) Proliferation (EdU incorporation for 8h) determined by flow cytometry in control Cre^{-} (n=2) or Cre^{+} *R3 fl/+* (n=1), and mutant Cre^{+} *R3 fl/fl* (n=5) P9 pups induced with Tamoxifen at P7 and P8. Horizontal lines represent mean. (**g**) Proliferation (EdU incorporation 14 h) determined by flow cytometry in E15 thoracic dorsal skin from control Cre^{-} (n=2) and mutant Cre^{+} *R3 fl/fl* (n=4) littermate embryos, induced by 3 consecutive i.p. injections of 4-OHT (1 mg) administered to the pregnant female (E12-E14). Horizontal lines represent mean. (**h**) Whole-mount immunofluorescence of ear skin of 3 weeks old mice injected intraperitoneally with PBS (control) or AAV-VEGFR3-Ig at P7. Note regression of dermal LYVE1^{+} lymphatic vessels (arrow) in the AAV-VEGFR3-Ig treated ear. * P<0.05, ** P<0.01, *** P<0.001. Two-tailed unpaired Student's *t*-test (**c**, **e-g**). Scale bars: 200 µm (**h**).

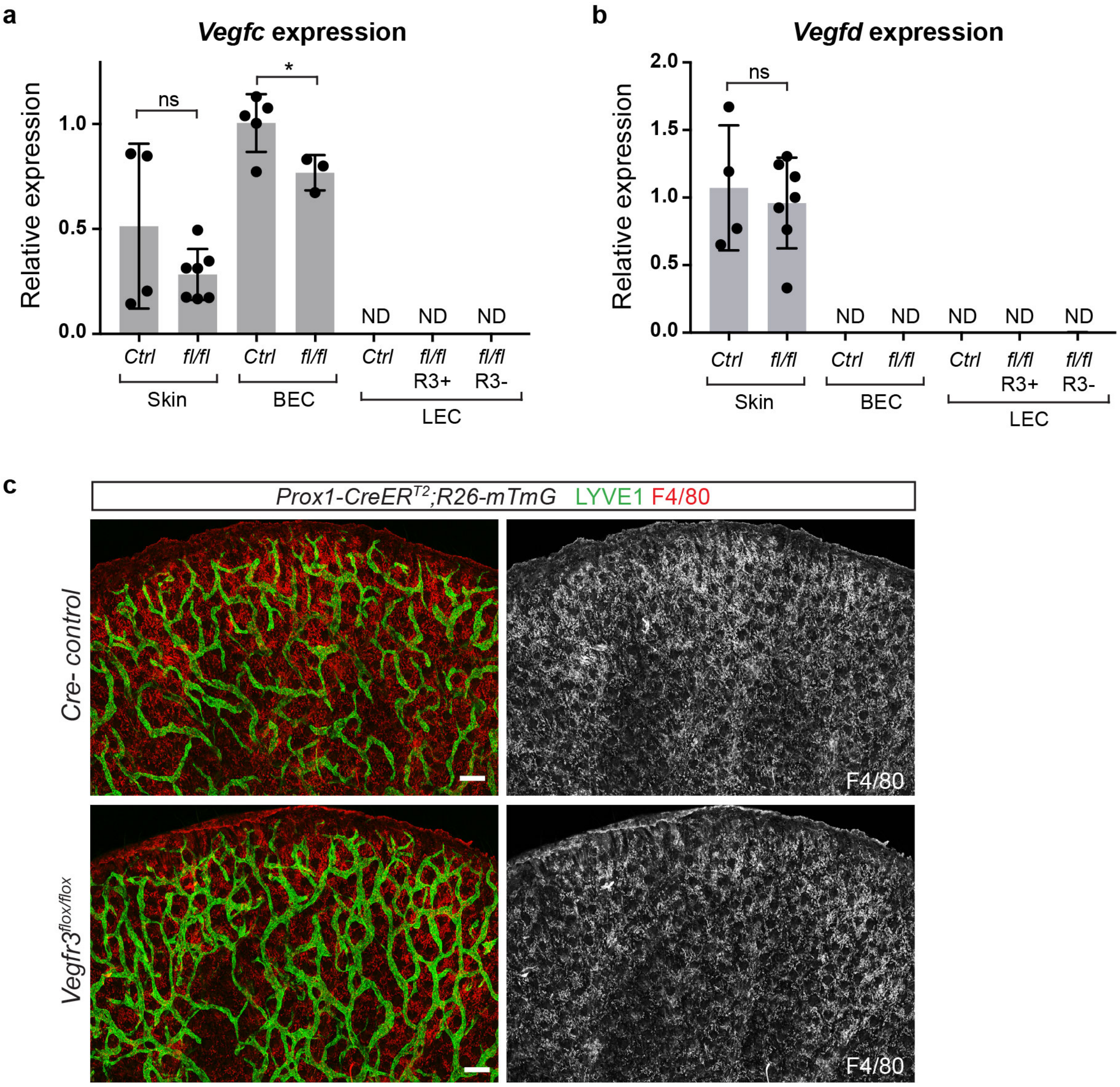

Supplementary Figure 7 | Analysis of VEGFC/D expression in the *Vegfr3*^{flox}*;R26-mTmG;Prox1-CreER*^{T2} skin. *Vegfc* (**a**) and *Vegfd* (*Figf*) (**b**) expression analysed by qRT-PCR in whole ear skin and FACS sorted BECs and LECs from P13-P14 *Vegfr3*$^{flox/flox}$*;R26-mTmG;Prox1-CreER*^{T2} (*fl/fl*) and Cre-negative (*Ctrl*) mice. Results from non-targeted (Tomato^{+}GFP^{-} ; R3^{+}), and targeted (Tomato^{-}GFP^{+} ; R3^{-}) LECs are shown separately. Bars represent mean relative expression, dots indicate individual mice (n=3-7 mice with n=2 technical replicates for each) ± s.d. ND, not detected. (**c**) Whole-mount immunofluorescence of ear skin of 3 weeks old *Vegfr3*$^{flox/flox}$*;R26-mTmG;Prox1-CreER*^{T2} and Cre-negative littermate mice treated with Tamoxifen at P2, P4 and P6. Single channel images for F4/80 staining are shown. * P<0.05, ns = not significant. Two-tailed unpaired Student's *t*-test (**a**, **b**). Scale bars: 200 μm (**c**).

Fig. 5d

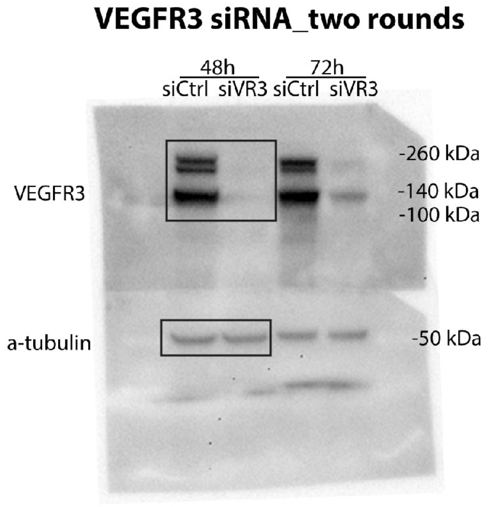

Fig. 7e

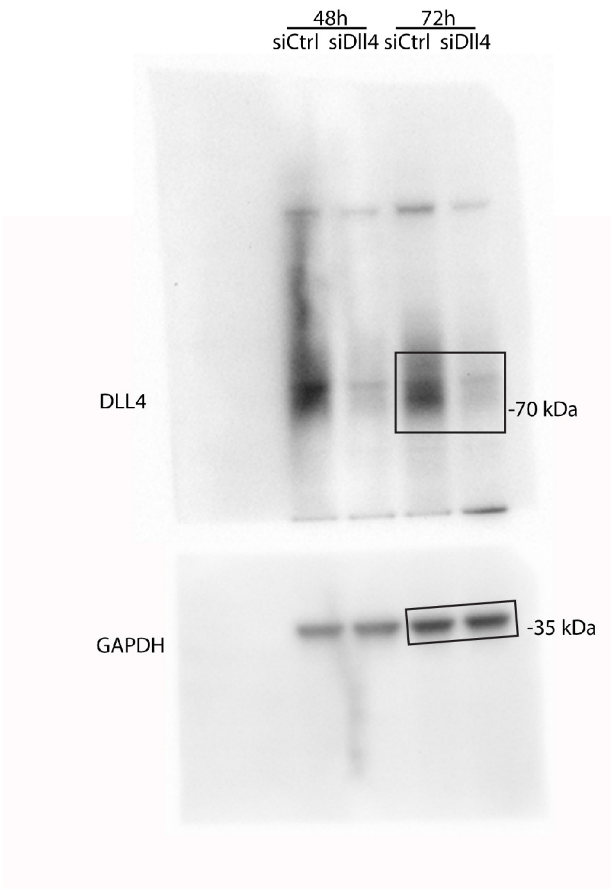

Fig. 7c

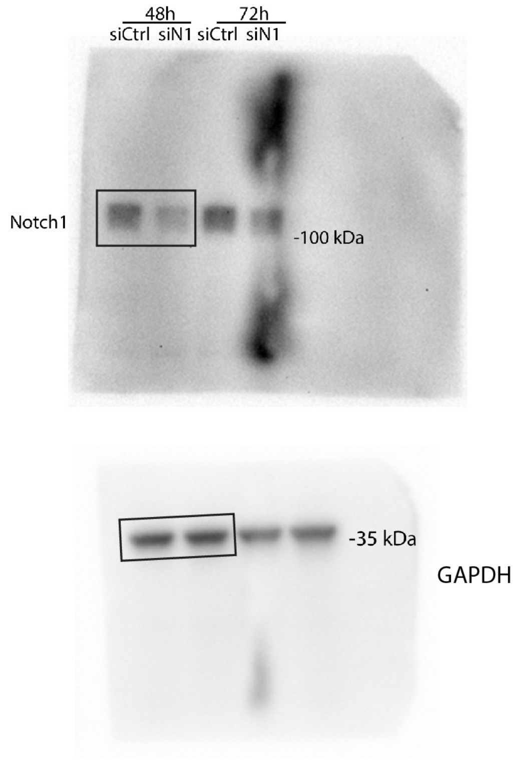

Supplementary Fig. 8 | Full Western blots. Portions of blots presented in Fig. 5d and Fig. 7c, e are indicated.
